# Upregulation of *ABLIM1* Differentiates Intrahepatic Cholangiocarcinoma from Hepatocellular Carcinoma and Both Colorectal and Pancreatic Adenocarcinoma Liver Metastases

**DOI:** 10.3390/genes15121545

**Published:** 2024-11-28

**Authors:** Tina Draškovič, Branislava Ranković, Nina Zidar, Nina Hauptman

**Affiliations:** Institute of Pathology, Faculty of Medicine, University of Ljubljana, 1000 Ljubljana, Slovenia; tina.draskovic@mf.uni-lj.si (T.D.); branislava.rankovic@mf.uni-lj.si (B.R.); nina.zidar@mf.uni-lj.si (N.Z.)

**Keywords:** hepatocellular carcinoma, cholangiocarcinoma, colorectal carcinoma, pancreatic ductal adenocarcinoma, colorectal liver metastases, pancreatic adenocarcinoma liver metastases, mRNA, lncRNA, gene expression, *ABLIM1*

## Abstract

Background: Altered gene expression in cancers holds great potential to improve the diagnostics and differentiation of primary and metastatic liver cancers. In this study, the expression of the protein-coding genes ring finger protein 135 (*RNF135*), ephrin-B2 (*EFNB2*), ring finger protein 125 (*RNF125*), homeobox-C 4 (*HOXC4*), actin-binding LIM protein 1 (*ABLIM1*) and oncostatin M receptor (*OSMR*) and the long non-coding RNAs (lncRNA) prospero homeobox 1 antisense RNA 1 (*PROX1-AS1*) and leukemia inhibitory factor receptor antisense RNA 1 (*LIFR-AS1*) was investigated in hepatocellular carcinoma, cholangiocarcinoma, colorectal liver metastases and pancreatic ductal adenocarcinoma liver metastases. Methods: This study included 149 formalin-fixed, paraffin-embedded samples from 80 patients. After RNA isolation, quantification, reverse transcription and preamplification, real-time qPCR was performed. The gene expression between different groups was calculated relative to the expression of the reference genes using the ∆∆Cq method and statistically analyzed. The expression of the genes was additionally analyzed using the AmiCA and UCSC Xena platforms. Results: In primary cancers, our results showed differential expression between primary tumors and healthy tissues for all the genes and lncRNA examined. Moreover, we found downregulation of *RNF135* in hepatocellular carcinoma, downregulation of *OSMR* in colorectal liver metastases and upregulation of *HOXC4* in cholangiocarcinoma compared to primary liver cancers and metastatic cancers. The major finding is the upregulation of *ABLIM1* in cholangiocarcinoma compared to hepatocellular carcinoma, colorectal liver metastases, pancreatic ductal adenocarcinoma liver metastases and healthy liver tissue. We propose *ABLIM1* as a potential biomarker that differentiates cholangiocarcinoma from other cancers and healthy liver tissue. Conclusions: This study emphasizes the importance of understanding the differences in gene expression between healthy tissues and primary and metastatic cancers and highlights the potential use of altered gene expression as a diagnostic biomarker in these malignancies.

## 1. Introduction

The majority of malignant diseases of the liver are adenocarcinomas, which consist of primary liver tumors and liver metastases. Since the liver is one of the most common sites of metastasis, metastatic liver adenocarcinomas are more common than primary liver tumors. The two most common primary adenocarcinomas of the liver are hepatocellular carcinoma (HCC), which arises from malignant transformation of hepatocytes, and cholangiocarcinoma (CCA), which arises from the epithelium of the bile ducts. As most cancers are very aggressive and are often detected at late stages, patients with HCC and CCA have a poor prognosis [1,2]. Due to the portal circulation, the liver is particularly susceptible to metastases of gastrointestinal origin. It is therefore not surprising that one of the most common liver metastases is from colorectal adenocarcinoma (CRC), which is the third most common malignancy worldwide [3]. Another common origin of metastases is pancreatic ductal adenocarcinoma (PDAC). Most PDAC patients have local progression or metastatic disease at the time of diagnosis [4]. The presence of liver metastases is associated with a poorer prognosis, as they continue to be a major cause of morbidity and mortality in CRC and PDAC patients.

The origin and differential diagnosis of liver adenocarcinomas can usually be determined by histomorphologic examination and the use of various established and routinely used immunohistochemical protein and molecular genetic markers. However, in some cases, primary liver adenocarcinomas are poorly differentiated and indistinguishable from metastatic adenocarcinomas [5,6]. HCC may have acinar structures or moderately to poorly differentiated features, making it difficult to differentiate metastatic adenocarcinoma from CCA [5,6]. Differentiation between CCA and metastatic PDAC is particularly difficult due to the similar morphologic pattern. In addition, both CCA and PDAC liver metastases (PCLMs) share immunohistochemical markers (e.g., MUC1 and CK19) and some driver mutations [7,8,9,10]. Without extensive accompanying clinical data, a definitive diagnosis is not possible. All this underscores the need for further research to identify more specific molecular markers that could aid in the diagnostic distinction between primary and secondary liver malignancies and reduce the diagnostic ambiguity. A better understanding of gene expression in these malignancies could lead to the identification of novel biomarkers that might improve the diagnostic sensitivity and specificity, particularly in the context of early detection.

Cancer cells often exhibit altered patterns of gene expression compared to normal cells. Common mechanisms that influence gene expression in cancer are epigenetic modifications (with DNA methylation being the most studied), non-coding RNAs (micro-RNAs and long non-coding RNAs (lncRNA)), genetic mutations and other genetic alterations [11,12,13,14]. Differences in the expression of oncogenes and tumor suppressor genes can contribute to the uncontrolled growth and progression of cancer. These genetic differences can be used to classify cancers, predict clinical outcomes and guide treatment decisions. In addition, the study of gene expression patterns in human adenocarcinomas can provide new insights into the pathophysiological pathways of cancer development, invasion and metastasis [15,16].

The main goal of our research was to investigate the expression of genes with potentially altered expression in common primary liver cancers and liver metastases. The genes included in our study were ring finger protein 135 (*RNF135*), ephrin-B2 (*EFNB2*), ring finger protein 125 (*RNF125*), homeobox-C 4 (*HOXC4*), actin-binding LIM protein 1 (*ABLIM1*), oncostatin M receptor (*OSMR*), lncRNA leukemia inhibitory factor receptor antisense RNA 1 (*LIFR-AS1*) and lncRNA prospero homeobox 1 antisense RNA 1 (*PROX1-AS1*). The genes included in this study were selected based on published work on bioinformatics and methylation analysis [17,18]. Briefly, we identified cancer-specific CpG methylation sites in gene promoters of the HM450 and EPIC platforms by differentially methylated regions analysis and assessed their methylation status experimentally by methylation-sensitive high-resolution melting and digital PCR. Based on the methylation bioinformatics data and experimental validation, a set of cancer-specific genes and/or lncRNAs were selected that differentiate each cancer type from other cancers and NATs: *RNF135* and *EFNB2* for HCC, *RNF125*, *ABLIM1* and *HOXC4* for CCA, *OSMR* and *LIFR-AS1* for CRC and CRC liver metastases (CRLM) and *PROX1-AS1* for PDAC and PCLM. This study explores the potential of altered expression of the investigated genes in the investigated cancer types to provide more refined and precise diagnostic markers that could complement existing immunohistochemical markers, particularly in cases where there is diagnostic ambiguity. To assess the potential differences in the expression of the selected genes, we performed gene expression analysis.

The aims of this study were to analyze the expression of six protein-coding genes and two lncRNAs in HCC, CCA, CRC, CRLM, PDAC, PCLM and normal tissues adjacent to tumors (NATs) and to investigate whether the expression differs between the included primary and metastatic liver adenocarcinomas and healthy liver tissue. Additionally, we investigated whether the expression of *OSMR* and *LIFR-AS1*, which are the genes whose methylation patterns are specific in differentiating CRC from other mentioned cancers, differs between primary CRC, CRC NATs and CRLM. Similarly, we investigated whether the expression of *PROX1-AS1*, whose methylation pattern is specific in differentiating PDAC from mentioned cancers, differs between primary PDAC, PDAC NATs and PCLM.

## 2. Materials and Methods

### 2.1. Study Population, Specimens and Ethics

This study included 149 formalin-fixed, paraffin-embedded (FFPE) resection samples from 80 patients. Histologic slides of HCC, intrahepatic CCA, CRC, PDAC, CRLM and PCLM collected between 2017 and 2023 were obtained from the archive of the Institute of Pathology, Faculty of Medicine, University of Ljubljana. Two certified pathologists performed the histopathological examination of the tissues and confirmed the diagnosis, tumor status and non-tumor tissue status. Of the primary tumors, a total of 15 HCCs, 15 CCAs, 15 CRCs and 15 PDACs were included in this study, along with their NATs. In addition, 19 CRLMs and 10 PCLMs were included. Paired samples of primary CRC, NATs and CRLM were obtained from 9 of the 19 CRLM patients. Paired samples from patients with PCLM were not available. The demographic data, including gender and age, with the disease category, histologic subtype, histologic grade and TNM classification of malignant tumors for all the patients are presented in Supplementary Table S1 [19,20,21]. The data included 45 men and 35 women. The average age of the patients was 66.8 years. This study was conducted in accordance with the Declaration of Helsinki and approved by the National Medical Ethics Committee of the Republic of Slovenia (No. 0120-34/2022/3).

### 2.2. RNA Isolation and Quantification

Tissue cores were punched from FFPE tissue blocks. RNA isolation from the punches was performed using the customized protocol on the Maxwell^®^ RSC Instrument (Promega Corporation, Madison, WI, USA). The protocol combined the Maxwell RSC FFPE Plus RNA Kit (Promega Corporation, Madison, WI, USA) and the Maxwell^®^ RSC miRNA Tissue Kit (Promega). The first part of the protocol was performed according to the Maxwell RSC FFPE Plus RNA Kit (Promega Corporation, Madison, WI, USA), which included deparaffinization of the samples, digestion with proteinase K and de-crosslinking of the RNA. The protocol was modified so that the protease digestion was performed overnight at 56 °C, mixing every 4 min for 15 s at 300 rpm. The next steps followed the instructions for the Maxwell^®^ RSC miRNA Tissue Kit (Promega Corporation, Madison, WI, USA). The lysis buffer and enhancer were added to the aqueous phase (skipping the 1-thioglycerol/homogenization solution step and proceeding to the next step). The entire lysate was added to the Maxwell RSC cartridge and extracted using the specific method. The total RNA was eluted in nuclease-free water. The quantification and quality of the RNA isolates were assessed with the Nanodrop spectrophotometer (ND-1000, Thermo Fisher Scientific, Waltham, MA, USA) and a Qubit fluorimeter (Thermo Fisher Scientific, Waltham, MA, USA) using the Qubit RNA HS Assay Kit (Thermo Fisher Scientific, Waltham, MA, USA). The ratios of the absorbance at the wavelengths of 230, 260 and 280 nm (A260/A280 and A260/A230) of all the included samples can be found in Supplementary Table S2.

### 2.3. Reverse Transcription and Real-Time Quantitative PCR

Reverse transcription (RT) reactions were performed with the High-Capacity cDNA Reverse Transcription Kit (Thermo Fisher Scientific) according to the manufacturer’s instructions on the SimpliAmp™ Thermal Cycler (Thermo Fisher Scientific, Waltham, MA, USA) with the following steps: 10 min at 25 °C, 120 min at 37 °C and 5 min at 85 °C. Here, 600 ng of RNA was added per RT reaction. After the cDNA synthesis, the quality of the samples and their suitability for our research was tested using the Hs_GAPDH_1_SG QuantiTect Primer Assay. The reactions were performed with the SYBR™ Select Master Mix according to the manufacturer’s instructions on the QuantStudio Real-Time PCR System (Thermo Fisher Scientific, Waltham, MA, USA). The standard cycling mode with its thermocycling conditions was 2 min at 50 °C, 2 min at 95 °C and 45 cycles of 15 s at 95 °C and 1 min at 60 °C.

For adequate samples, the preamplification reaction was performed using the TaqMan PreAmp Master Mix Kit (Thermo Fisher Scientific, Waltham, MA, USA) according to the manufacturer’s protocol. The SimpliAmp™ Thermal Cycler (Applied Biosystems, Thermo Fisher Scientific) and the ProFlex PCR System (Thermo Fisher Scientific, Waltham, MA, USA) were used for the incubation steps with enzyme activation for 10 min at 95 °C, 14 cycles of annealing and extension for 15 s at 95 °C and 5 min at 60 °C and enzyme inactivation for 10 min at 99 °C. The final preamplification reaction was diluted 20× according to the manufacturer’s recommendations.

The real-time qPCR reactions for the selected genes were performed using TaqMan technology with the FastStart Essential DNA Probe Master (Roche, Basel, Switzerland) on the QuantStudio Real-Time PCR System (Thermo Fisher Scientific, Waltham, MA, USA). The thermocycling conditions were 2 min at 50 °C, 10 min at 95 °C and 45 cycles of 15 s at 95 °C and 1 min at 60 °C. The TaqMan assays (Thermo Fisher Scientific, Waltham, MA, USA) used are listed in Table 1. The samples were run in duplicate, whereas the qPCR efficiency reactions were run in triplicate. Pools of RNA samples from each group were created to calculate the efficiency of the qPCR reactions. A two-fold serial dilution of each pool was prepared in six steps. The dilutions ranged from 20- to 640-fold dilutions.

### 2.4. Reference Genes Selection

Candidate reference genes were selected from the literature, including *GAPDH*, *B2M* and *IPO8* (Table 1) [22,23,24,25]. To evaluate the stability of the candidate reference genes in our samples and to determine the most stable pair of reference genes, we used the geNorm algorithm, which is included in the ctrlGene package (version 1.0.1) in the R software environment (version 4.3.0). For each reference gene, the algorithm calculated the M value, a measure of the expression stability based on the average pairwise expression ratio, and suggested the best gene pair and calculated its M value. The gene or combination of genes with the lowest M value was considered the most stable [26].

### 2.5. Validation with Publicly Available Online Tools

To further corroborate our experimental findings, we searched for publicly available data and used tools for data collection, visualization and interpretation. To obtain data on the differential expression of the studied genes in our primary cancers compared to paired healthy tissues, we used the tool AmiCA [27]. Using the ‘Expression by disease’ tab, we obtained an overview of the log2 fold changes of the investigated genes across the project from the TCGA database. Furthermore, we collected data for the investigated genes and lncRNA from the UCSC Xena platform [28]. We collected Illumina HiSeq data and visualized the expression for individual genes in different primary cancers and healthy tissues with boxplots. The data included 50 HCC NAT, 371 HCC, 9 CCA NAT, 36 CCA, 51 CRC NAT, 380 CRC, 4 PDAC NAT and 178 PDAC samples. We calculated the mean of the log2(norm_count+1) values for each group. The relative changes in the expression levels, the fold change (FC) and statistics between groups were calculated. Data for CRLM and PCLM were not available for either platform.

### 2.6. Statistical Analysis

The gene expression analysis was performed to test whether the expression of the selected genes was statistically significantly upregulated or downregulated between primary tumors, metastases and healthy liver tissue. The gene expression between the different groups was calculated relative to the expression of the two reference genes using the ∆∆Cq method [29]. The ∆Cq data were obtained by normalizing to the geometric mean of the *B2M* and *IPO8* reference genes for the mRNAs and lncRNAs studied. A higher ΔCq indicates a lower mRNA abundance of the target gene, whereas lower ΔCq values indicate a higher mRNA abundance of the target gene.

IBM SPSS Statistics 27 was used for the statistical analysis of the experimental results and the results of the Xena platform. Kolmogorov–Smirnov and Shapiro–Wilk tests were used to check the normal distribution of the experimental data. If the data were normally distributed, parametric tests (paired *t*-tests for dependent samples and *t*-tests for independent samples) were used. If the data were not normally distributed, non-parametric tests (Wilcoxon signed-rank test for dependent samples and Mann–Whitney U test for independent samples) were used.

## 3. Results

### 3.1. Reference Genes

**Figure 1:**
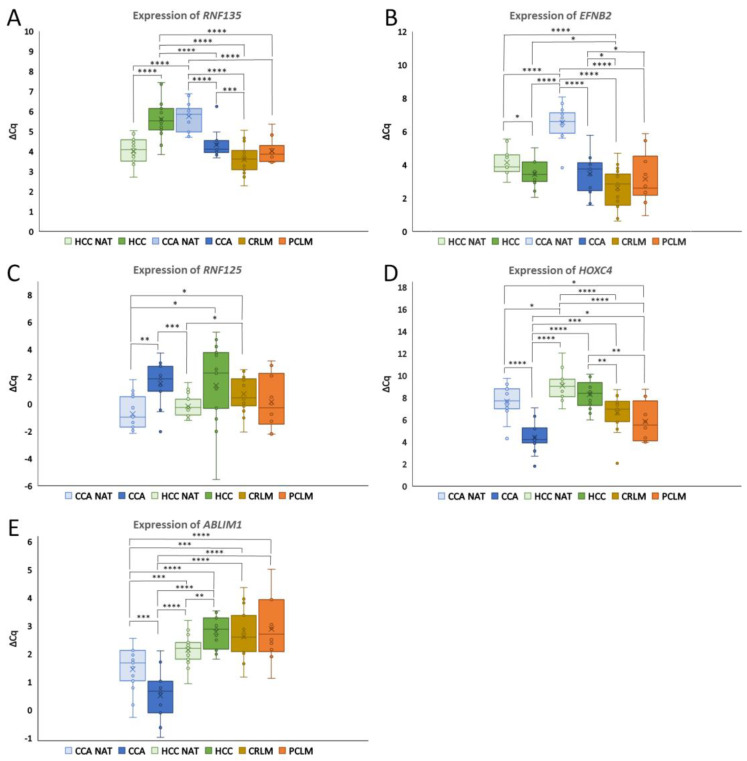
Expression of (**A**) *RNF135*, (**B**) *EFNB2*, (**C**) *RNF125*, (**D**) *HOXC4* and (**E**) *ABLIM1* in HCC, HCC NATs, CCA, CCA NATs, CRLM and PDLM. The expression is presented as the ∆Cq values and the significance between the different groups is shown. The whiskers of the boxplots represent the values of the upper and lower quartiles and the dots represent outliers. The line dividing the boxplot represents the median and the cross represents the mean. Legend: * *p* ≤ 0.05, ** *p* ≤ 0.01 *** *p* ≤ 0.001, **** *p* ≤ 0.0001; Cq, quantification cycle; HCC NAT, normal liver tissue adjacent to hepatocellular carcinoma; HCC hepatocellular carcinoma, CCA NAT, normal liver tissue adjacent to cholangiocarcinoma; CCA, cholangiocarcinoma; CRLM, colorectal liver metastases; PCLM, pancreatic ductal adenocarcinoma liver metastases.

**Figure 2:**
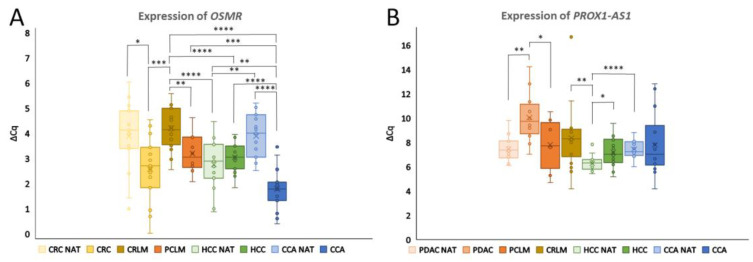
Expression of *OSMR* and *PROX1-AS1*. The expression is plotted as the ∆Cq values and the significance between different groups is presented. The whiskers of the boxplots represent the values of the upper and lower quartiles and the dots represent the outliers. The line dividing the boxplot represents the median and the cross represents the mean. (**A**) *OSMR* expression in CRC, CRC NAT, CRLM, PCLM, HCC, HCC NAT, CCA and CCA NAT. (**B**) *PROX1-AS1* expression in PDAC, PDAC NAT, PCLM, CRLM, HCC, HCC NAT, CCA and CCA NAT. Legend: * *p* ≤ 0.05, ** *p* ≤ 0.01 *** *p* ≤ 0.001, **** *p* ≤ 0.0001; Cq, quantification cycle; HCC NAT, normal liver tissue adjacent to hepatocellular carcinoma; HCC hepatocellular carcinoma, CCA NAT, normal liver tissue adjacent to cholangiocarcinoma; CCA, cholangiocarcinoma; CRC NAT, normal colon tissue adjacent to colorectal cancer; CRC, colorectal carcinoma; CRLM, colorectal liver metastases; PDAC NAT, normal pancreatic tissue adjacent to pancreatic ductal adenocarcinoma; PDAC, pancreatic ductal adenocarcinoma; PCLM, pancreatic ductal adenocarcinoma liver metastases.

The gene expression (∆Cq) was calculated relative to the expression of the two reference genes and used to calculate the FC of the group comparisons. The stability and suitability of the candidate reference genes for all the included healthy and cancerous tissues was confirmed using the geNorm algorithm [26]. The stability of the reference genes in all the included primary tumors, liver metastases and NATs, expressed with the M value for *GAPDH*, *B2M* and *IPO8*, was 1.27, 1.16 and 1.02, respectively. The highest stability of 0.92 was achieved for the *B2M*–*IPO8* pair, which was then selected as the most suitable reference gene pair and used for the following calculations. Using the ∆∆Cq method, we compared the expression between the groups [29]. The ∆Cq values of each group for the genes analyzed are shown in Figure 1 and Figure 2. The ∆∆Cq, FC and *p*-values of all the included comparisons can be found in Supplementary Table S3.

### 3.2. Differential Expression in Primary Cancers Compared to Paired Healthy Tissues

The first part of our research focused on whether the studied genes were differentially expressed between the primary tumors for which they were identified and their NATs (*RNF135* and *EFNB2* for HCC, *RNF125*, *ABLIM1* and *HOXC4* for CCA, *OSMR* and *LIFR-AS1* for *OSMR* and *PROX1-AS1* for PDAC). We found differential expression between the primary tumors and healthy tissues for all the genes and lncRNA examined. In HCC, we focused on *RNF135* and *EFNB2*. When comparing HCC with HCC NATs, *RNF135* was downregulated (FC = −2.97, *p* < 0.0001) and *EFNB2* was upregulated (FC = 1.63 and *p* < 0.05) (Figure 1A,B). Interestingly, both genes also showed pronounced upregulation in CCA compared to CCA NATs. Specifically, *RNF135* was upregulated by 2.68-fold (*p* < 0.0001) and *EFNB2* showed an even larger increase of 8.41-fold (*p* < 0.0001).

In CCA, the genes analyzed included *RNF125*, *HOXC4* and *ABLIM1*. *RNF125* was downregulated (FC = −4.69, *p* < 0.01), while *ABLIM1* and *HOXC4* were upregulated in CCA compared to CCA NATs (Figure 1C–E). The *ABLIM1* expression was 1.92-fold higher (*p* < 0.001) and the *HOXC4* expression was 10.02-fold higher in CCA than in CCA NATs (*p* < 0.0001). In contrast, in HCC, *ABLIM1* was the only gene to show a significant difference compared to HCC NATs, with a downregulation of 1.56-fold (*p* < 0.01).

In CRC, upregulation of *OSMR* was observed in CRC compared to CRC NATs (FC = 2.49, *p* < 0.05) (Figure 2A). Additionally, *OSMR* expression differed significantly in CCA, where it was 4.27-fold higher than in CCA NATs (*p* < 0.0001).

Downregulation of lncRNA *PROX1-AS1* was observed in PDAC compared to PDAC NATs (FC = −4.96, *p* < 0.01) (Figure 2B). Moreover, a significant reduction in the *PROX1-AS1* expression was also noted in HCC versus HCC NATs, with a fold change of −1.87 (*p* < 0.05).

### 3.3. Differential Expression Between Primary Liver Cancers, Liver Metastases and Healthy Liver Tissue

The second part of our research focused on whether the studied genes and lncRNA were differentially expressed between the primary CRC and PDAC tumors and their metastases and whether the expression of these genes differed between the primary liver cancers, CRLM and PDAC.

From our analysis, three genes emerged as potential biomarkers for differentiating CCA from other groups, with *ABLIM1* being the most promising candidate. *ABLIM1* was significantly upregulated in CCA compared to all the other groups, making it the most distinctive gene for differentiating CCA from both HCC and common liver metastases (Figure 1E). Specifically, the *ABLIM1* expression in CCA was upregulated by 1.92-fold compared to CCA NATs (*p* < 0.001), 3.10-fold compared to HCC NATs (*p* < 0.0001), 4.83-fold compared to HCC (*p* < 0.0001), 4.72-fold compared to CRLM (*p* < 0.0001) and 5.20-fold compared to PCLM (*p* < 0.0001). This strong differential expression of *ABLIM1* indicates it to be a promising candidate as a biomarker for differentiating CCA from both primary liver cancers and metastatic liver cancers.

Another promising gene for differentiating CCA was *HOXC4* (Figure 1D). *HOXC4* was highly upregulated in CCA compared to CCA NATs (*p* < 0.0001), showing a 25.70-fold increase compared to HCC NATs (*p* < 0.0001), a 19.96-fold increase compared to HCC (*p* < 0.0001), a 4.31-fold increase compared to CRLM (*p* < 0.001) and a 2.73-fold increase compared to PCLM (*p* < 0.05).

*OSMR* also demonstrated strong potential for differentiating CCA from other groups (Figure 2A). *OSMR* was upregulated in CCA compared to CCA NATs, as previously discussed, and showed significant upregulation compared to HCC (FC = 2.39, *p* < 0.0001), HCC NATs (FC = 1.96, *p* < 0.01), PCLM (FC = 2.65, *p* < 0.001) and CRLM (FC = 5.33, *p* < 0.0001). Additionally, OSMR was downregulated in CRLM compared to CRC (FC = −3.10, *p* < 0.001), although no significant difference was found between CRLM and CRC NATs.

*RNF135* was identified as a promising marker for differentiating HCC from liver metastases (Figure 1A). *RNF135* was significantly downregulated in HCC compared to CCA, CRLM and PCLM (FC = −2.42, FC = −3.95, FC = −2.99, all *p* < 0.0001). However, there was no significant difference in the RNF135 expression between HCC and CCA NATs, suggesting a tumor-specific downregulation in HCC.

*EFNB2* exhibited the lowest expression in CCA NATs and was consistently upregulated in all the other groups when compared to CCA NATs (Figure 1B).

*RNF125* had the lowest expression in primary liver tumors (Figure 1C). A significant downregulation of *RNF125* was observed in CCA compared to HCC NATs (FC = −3.19, *p* < 0.001) and a significant difference was also observed between HCC and CCA NATs (FC = −4.22, *p* < 0.05). *RNF125* was additionally downregulated in CRLM compared to healthy liver tissue.

Lastly, the lncRNA *PROX1-AS1* exhibited differential expression between PDAC, PDAC NATs and PCLM. *PROX1-AS1* was upregulated in PCLM compared to PDAC (FC = 4.09, *p* < 0.05), although no significant difference was observed between PCLM and PDAC NATs (Figure 2B).

### 3.4. Additional Validation with AmiCA and USCS Xena Platforms

**Figure 3:**
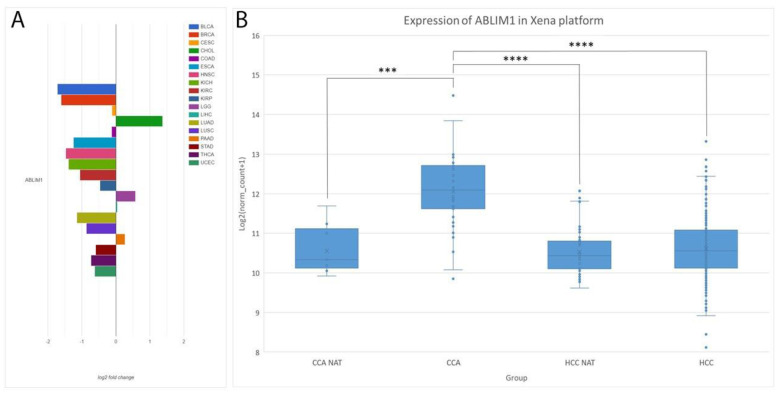
Expression of *ABLIM1* in the AmiCA and Xena platforms. (**A**) *ABLIM1* expression in TCGA projects on the AmiCA platform. The log2 fold change between cancer and NAT for each cancer type is shown. (**B**) *ABLIM1* expression in the Xena platform. The expression is plotted as the log2(norm_count+1) values and the significance between different groups is presented. The whiskers of the boxplots represent the values of the upper and lower quartiles and the dots represent the outliers. The line dividing the boxplot represents the median and the cross represents the mean. Legend: *** *p* ≤ 0.001, **** *p* ≤ 0.0001; HCC NAT, normal liver tissue adjacent to hepatocellular carcinoma; HCC hepatocellular carcinoma, CCA NAT, normal liver tissue adjacent to cholangiocarcinoma; CCA, cholangiocarcinoma.

To support our experimental results, we used two publicly available tools: the AmiCA and Xena platforms [27,28]. We used data from the primary cancers as data for CRLM and PCLM were not available. Using the AmiCA tool, we obtained an overview of the differential expression of the investigated genes in primary liver cancers compared to paired healthy tissues. Similarly, we have collected and statistically analyzed data for 1079 primary cancer and healthy tissue samples from the Xena platform. The results from the AmiCA and Xena platforms for *RNF135*, *EFNB2*, *RNF125*, *HOXC4*, *OSMR* and *PROX1-AS1* are presented in Supplementary Figure S1. Here, we focused on the best-performing gene, *ABLIM1* (Figure 3). The AmiCA results confirm the upregulation of *ABLIM1* in CCA compared to healthy liver tissue (Figure 3A). Taking an overview of the different cancers, we can see that *ABLIM1* is downregulated in the majority of other cancers, including HCC, which is consistent with our experimental results (Figure 1E). For the *ABLIM1* analysis from the Xena platform, we included 466 samples of CCA, CCA NAT, HCC and HCC NAT samples. The results showed a 2.82-fold upregulation of *ABLIM1* in CCA compared to CCA NATs (*p* < 0.001). The expression of *ABLIM1* was also upregulated in CCA compared to HCC and HCC NATs (FC = 2.70, *p* < 0.0001 and FC = 2.87, *p* < 0.0001, respectively) (Figure 3B).

### 3.5. Gene Functions

To gain a deeper understanding of the role of specific genes and lncRNAs in cancer development, we conducted a comprehensive literature review. Supplementary Table S4 provides an overview of the investigated genes and lncRNAs and describes their functions and involvement in cancer. These genes and lncRNAs contribute to cancer progression by regulating key processes such as cell proliferation, migration, invasion, epithelial–mesenchymal transition and apoptosis. Some also influence angiogenesis, alter the tumor microenvironment and enhance tumor immunity by inhibiting immune cell infiltration. In addition, some of them modulate ubiquitination and proteasome-mediated degradation. They influence important oncogenic signaling pathways such as the PI3K/AKT/mTOR, MAPK/ERK, Wnt/β-catenin and TGF-β1-SMAD3-ID1 signaling pathways [30,31,32,33,34,35,36,37,38,39,40,41,42,43,44,45,46,47,48,49,50,51,52,53,54,55,56,57,58,59,60,61,62,63,64,65,66].

## 4. Discussion

Comparison of the gene expression profiles of primary and metastatic liver cancers has great potential to increase diagnostic accuracy. Organ-specific and cancer-specific gene expression patterns can aid in the diagnosis of liver cancer. Studies on gene expression in liver metastases from CRC successfully demonstrate the potential of this approach to differentiate between primary liver cancer and liver metastases, with an accuracy of over 90% in identifying the primary tumor sites [16]. Incorporating gene expression assays into clinical practice will allow oncologists to offer precise treatments, improving care and outcomes for patients with primary and metastatic liver cancers. In addition, understanding the differences in expression between CRLM, PCLM and primary liver cancers (HCC and CCA) may provide insights into their distinct biological behavior, indicating potential therapeutic targets and prognostic markers.

Our experimental results (Figure 1 and Figure 2) show altered gene expression of the investigated genes between HCC, CCA, CRLM, PCLM and healthy liver tissue and are therefore a promising additional tool to differentiate between primary liver cancers and liver metastases, which is in line with the literature data [16]. Furthermore, additional validation with the AmiCA and USCS Xena platforms showed agreement with our experimental results.

The major finding of our study is the upregulation of *ABLIM1* in CCA compared to HCC, CRLM, PCLM and healthy liver tissue. *ABLIM1*, the expression of which had not previously been studied in CCA, was upregulated and may also serve as a potential biomarker for CCA. Other studies showed upregulation of ABLIM1 in CRC patients and demonstrated its function in promoting tumor growth and metastasis in vitro [55]. In HCC, high expression of ABLIM1 was associated with a poor prognosis [54].

Similarly, our results show significantly higher expression of *HOXC4* in CCA compared to the other groups, suggesting its potential role as a biomarker. Careful interpretation is necessary, as HOXC4 has been identified as an oncogene in various cancers. Higher expression was found in cancer tissues compared to normal tissues in 21 tumor types, including HCC, CCA, CRC and PDAC, which is consistent with our experimental results [53].

Our findings reveal variable *OSMR* expression across cancer types: it is elevated in CRC compared to CRC NATs but downregulated in CRLM. It may act as a tumor suppressor and its low expression has been associated with resistance to tumor growth inhibition [62]. Notably, the *OSMR* levels were lowest in CRLM compared to CCA, CRLM and PCLM, indicating *OSMR* could serve as a biomarker to differentiate CRLM from other cancers. Targeted therapies that block OSMR or its ligand OSM are currently being explored [58,59,60,61].

Another important finding is the significant downregulation of *RNF135* in HCC compared to CCA, CRLM and PCLM, suggesting that its lower expression is characteristic of HCC and may help to differentiate HCC from other cancers. Our results confirm Wang’s findings that *RNF135* is downregulated in HCC, where it acts as a tumor suppressor and is linked to a poor prognosis [32]. The high expression of *RNF135* in CRLM is aligned with the Qiu et al.’s report of *RNF135* upregulation in CRC [35]. It is also associated with chemotherapy resistance [35]. As DNA methylation regulates *RNF135* expression, patients with low *RNF135* expression may benefit from demethylation drugs, potentially improving their clinical outcomes [32]. Our previous study also revealed hypermethylation of the *RNF135* promoter, which may explain the reduced *RNF135* expression observed in our samples and additionally support the role of methylation in regulating *RNF135* expression [17].

*RNF125* expression is significantly downregulated in several cancers, including CCA, HCC and CRC, and is negatively correlated with the clinical stage, whereas higher expression is associated with better clinical outcomes [38,39,42]. Our results confirm this, as RNF125 was downregulated in HCC and CCA compared to healthy liver tissue. Furthermore, the hypermethylation of the *RNF125* promoter in CCA discovered in our previous study may explain its reduced expression, as promoter hypermethylation has also been associated with its downregulation in other diseases [17,36]. *RNF125* was also downregulated in CRLM in comparison to healthy liver tissue, which may be consistent with the downregulation of *RNF125* in primary CRC [42]. RNF125 downregulation is associated with a poor prognosis and disease progression [39,41].

*EFNB2* has been linked to HCC progression, with significantly higher expression in HCC compared to normal tissue, which our results confirm [43,44]. Similarly, *EFNB2* is upregulated in CCA and CRLM compared to normal liver tissue, which aligns our results with previous findings [45,49]. Other studies also reported overexpression in CRC samples [48,50]. In PDAC, *EFNB2* is overexpressed and associated with tumor progression, making it a potential treatment target [47].

We investigated the expression of the lncRNA *PROX1-AS1*, which is involved in tumor growth and metastasis in some malignancies [63,64,65]. Our results revealed *PROX1-AS1* downregulation in PDAC and HCC compared to their NATs, but its expression increased from PDAC to PCLM. In CRC and other cancers, *PROX1-AS1* expression is upregulated and high *PROX1-AS1* expression is associated with poor overall survival [63].

Another interesting finding is that the expression of some of the investigated protein-coding genes and lncRNAs differs between HCC NATs and CCA NATs (Figure 1 and Figure 2). These differences may stem from the distinct cellular origins and functions: hepatocytes for HCC and biliary epithelial cells (cholangiocytes) for CCA. In HCC, the adjacent tissue may show alterations related to liver metabolism and regeneration, whereas in CCA, the surrounding tissue may show changes in gene expression related to bile duct function and inflammation [67,68]. To better understand the observed differences, the expression of our mRNAs was compared to the protein expression data from the Human Protein Atlas, which is based on conventional immunohistochemistry [69]. Comparing the mRNA levels with the protein expression, we found consistency for RNF135 and EFNB2 but some discrepancies for ABLIM1 and OSMR. The protein expression of RNF125 was not detectable in either hepatocytes or cholangiocytes. Data for HOXC4 were not available. Differences may arise due to post-transcriptional regulation or molecular changes influenced by proximity to tumors, positioning NAT tissue as an intermediate between the healthy and malignant states [70,71]. Further studies are needed to understand the differences in expression between HCC NATs and CCA NATs.

The limitation of our study is the small sample size for individual cancers. Another technical limitation of this study is the low detection limit of expression by real-time qPCR. In addition to the presented genes and lncRNA, another lncRNA, *LIFR-AS1*, was investigated in this study. We were able to determine its expression in less than half of the healthy colon tissue and only in one CRC sample. We can assume that *LIFR-AS1* was downregulated, so that its expression was below the detection limit in the majority of samples. Regarding the bioinformatics validation of our experimental results, we found that there are no data for CRLM and PCLM in the publicly available databases and platforms. If available, additional data on the expression profiles of metastases would allow further confirmation of our experimental results. Another limitation is the lack of in situ analysis of the diagnostic biomarkers used at the protein level by immunohistochemistry.

This study demonstrates the possibility of using signature genes to differentiate between primary liver cancer, liver metastases and healthy liver tissue. A large-scale clinical study with a larger number of patients is needed to further substantiate our results.

## 5. Conclusions

This study expands our understanding of the transcriptomic behavior of *RNF135*, *EFBN2*, *RNF125*, *ABLIM1*, *HOXC4*, *OSMR* and *PROX1-AS1* in HCC, CCA, CRLM, PCLM and NATs. They have unique and overlapping mRNA and lncRNA expression profiles. Our results show differential expression between primary tumors and healthy tissues for all the genes and lncRNAs examined. Our most important finding is the significantly higher expression of *ABLIM1* compared to healthy liver tissue and other cancers. Therefore, *ABLIM1* has the potential to differentiate CCA from HCC, CRLM, PCLM and healthy liver tissue. In addition, significantly higher expression of *HOXC4* was detected in CCA, significantly lower expression of *RNF135* in HCC and significantly lower expression of *OSMR* in CRLM compared to the other cancers. In conclusion, comparing the gene expression profiles of primary and metastatic liver cancer holds the potential to identify diagnostic biomarkers, increase diagnostic accuracy and identify potential therapeutic targets.

## Figures and Tables

**Table 1:** List of used TaqMan assays (Thermo Fisher Scientific).

mRNA or lncRNA Gene	Assay ID
*GAPDH*	Hs03929097_g1
*B2M*	Hs99999907_m1
*IPO8*	Hs00183533_m1
*RNF135*	Hs00810675_m1
*EFNB2*	Hs00187950_m1
*RNF125*	Hs00215201_m1
*HOXC4*	Hs00538088_m1
*ABLIM1*	Hs01046520_m1
*OSMR*	Hs01055340_m1
*LIFR-AS1*	Hs01373895_m1
*PROX1-AS1*	Hs01368902_m1

## Data Availability

The original contributions presented in this study are included in the article. Further inquiries can be directed to the corresponding author upon reasonable request.

## Supplementary Materials

The following supporting information can be downloaded at https://www.mdpi.com/article/10.3390/genes15121545/s1. Table S1: The demographic data, including gender and age, disease type, histologic subtype, histologic grade and TNM classification of malignant tumors. TNM staging was performed in accordance with the UICC 8th edition 2017. Histological grading was performed using the standard grading system according to the WHO Classification of Tumors (well, moderately or poorly differentiated) and a classification based on the histopathological criteria focusing on the structural and cytological features of the tumor into one of the two recommended groups for mucinous adenocarcinomas (differentiated or undifferentiated). Legend: M, men. F, female. HCC, hepatocellular carcinoma. CCA, cholangiocarcinoma. PDAC, pancreatic ductal adenocarcinoma. PCLM, pancreatic ductal adenocarcinoma liver metastases. CRC, colorectal adenocarcinoma. CRLM, colorectal liver metastases. NOS, not otherwise specified. Table S2: Nanodrop measurements. Included are the ratios of absorbance at the wavelengths of 230, 260 and 280 nm (A260/A280 and A260/A230) of all the included samples. Abbreviations: HCC, hepatocellular carcinoma; CCA, cholangiocarcinoma; PDAC, pancreatic ductal adenocarcinoma; PCLM, pancreatic ductal adenocarcinoma liver metastases; CRC, colorectal adenocarcinoma; CRLM, colorectal liver metastases; NAT, normal tissue adjacent to tumor. Table S3: The ∆∆Cq, FC and *p*-values of all the included comparisons. The statistically significant results are highlighted in bold. Abbreviations: HCC, hepatocellular carcinoma; CCA, cholangiocarcinoma; PDAC, pancreatic ductal adenocarcinoma; PCLM, pancreatic ductal adenocarcinoma liver metastases; CRC, colorectal adenocarcinoma; CRLM, colorectal liver metastases; NAT, normal tissue adjacent to tumor. Table S4: An overview of the included genes and lncRNAs with a description of their functions, their involvement in cancer and their involvement in cancer-related signaling pathways. Figure S1: The results of the analyses of the AmiCA and Xena platforms for *RNF135*, *EFNB2*, *RNF125*, *HOXC4*, *OSMR* and *PROX1-AS1*. For the lncRNA *PROX1-AS1*, the data were sourced solely from the Xena platform, as the AmiCA platform does not contain lncRNA data. From the Xena platform, we included 1079 samples of HCC, HCC NAT, CCA, CCA NAT, CRC, CRC NAT, PDAC and PDAC NAT samples. Abbreviations: HCC, hepatocellular carcinoma; CCA, cholangiocarcinoma; PDAC, pancreatic ductal adenocarcinoma; CRC, colorectal adenocarcinoma; NAT, normal tissue adjacent to tumor; * *p* ≤ 0.05, ** *p* ≤ 0.01; *** *p* ≤ 0.001, **** *p* ≤ 0.0001.
